# Gastric Acid-Protective and Intestinal Targeted Nanogels Enable Anti-Bacterial Activity of Cefquinome

**DOI:** 10.3390/gels11070503

**Published:** 2025-06-27

**Authors:** Xianqiang Li, Tianhui Wang, Shuo Han, Jinhuan Liu, Xiuping Zhang, Zhiqiang Zhou, Ali Sobhy Dawood, Wanhe Luo

**Affiliations:** 1 College of Biomedicine and Health, Anhui Science and Technology University, Chuzhou 233100, China; lixianqiang89@sina.com; 2 Engineering Laboratory for Tarim Animal Diseases Diagnosis and Control, College of Animal Science and Technology, Tarim University, Alar 843300, China; w3505506223@163.com (T.W.); 15645977635@163.com (S.H.); liujinhuan0830@163.com (J.L.); zxpkdy@126.com (X.Z.); 3 College of Veterinary Medicine, Sichuan Agricultural University, Chengdu 611130, China; 4 Instrumental Analysis Center, Tarim University, Alar 843300, China; zhou2011519@sina.com; 5 Infectious Diseases, Faculty of Veterinary Medicine, University of Sadat City, Sadat City 32897, Egypt; ali.dawood@vet.usc.edu.eg

**Keywords:** cefquinome (CEF), *Escherichia coli*, sustained release and targeted release, carboxymethylcellulose sodium (CMCNa), D-mannosamine hydrochloride (DMH)

## Abstract

To enhance the antibacterial activity of cefquinome (CEF) against *Escherichia coli*, a Carboxymethylcellulose sodium (CMCNa)/D-Mannosamine hydrochloride (DMH)-based nanogels delivery system capable of protecting CEF from gastric acid degradation while enabling intestinal sustained release and targeted antibacterial enhancement was developed. Systematic research was conducted on the best formulation, physicochemical characteristics, stability, gastrointestinal fluid-responsiveness, and antibacterial activity of the optimal formulation. The results showed that the optimized CEF nanogels demonstrated an enhanced loading capacity (13.0% ± 1.7%) and encapsulation efficiency (52.2% ± 1.0%). CEF nanogels appeared as uniform transparent spheres with a smooth surface under transmission electron microscopy and exhibited a three-dimensional porous network via scanning electron microscopy. More importantly, stability studies revealed that the CEF nanogels hold satisfactory stability. In addition, the formed CEF nanogels could effectively avoid the destruction of CEF by gastric acid in simulated gastric juice. In addition, they had the effect of slow and targeted release in the simulated intestinal tract. Compared to the free CEF, CEF nanogels have stronger antibacterial activity against *Escherichia coli*. In short, the prepared CEF nanogels had stronger antibacterial activity than CEF through sustained and targeted release.

## 1. Introduction

Animal-derived diarrhea, predominantly caused by pathogenic *Escherichia coli* (*E. coli*), is one of the most prevalent diseases in the livestock industry. This disease induces growth retardation, reduced production performance, and results in high mortality rates, leading to substantial economic losses in animal husbandry [1,2,3]. Currently, antibiotic therapy remains the primary approach for treating *E. coli*-associated diarrhea [4]. Cefquinome sulfate (CEF), a fourth-generation cephalosporin, has a broad-spectrum antibacterial action, low toxicity, and good stability, which has led to its widespread use in veterinary clinics [5]. The bactericidal effect of CEF is achieved through the inhibition of bacterial cell wall synthesis, demonstrating remarkable efficacy against Gram-negative bacteria, such as *E. coli* [6]. However, significant limitations hinder its application in veterinary clinic: (1) oral administration of CEF is compromised by gastric acid degradation, drastically reducing its bioavailability; (2) conventional formulations lack sustained-release and targeted delivery capabilities, resulting in insufficient drug accumulation at infection sites, which not only diminishes therapeutic efficacy, but also increases the risk of bacterial resistance due to frequent administration [7,8]. Consequently, the development of a drug delivery system capable of protecting CEF from gastric acid degradation, while enabling sustained and targeted delivery, is urgently required to enhance its antimicrobial performance.

More and more nanocarrier systems are being used to enhance the antibacterial activity of CEF, such as CEF oily nanosuspension, CEF proliposomes, and CEF gelatin microspheres [5,7,8]. The larger particle size, the lower loading capacity (LC), and the reduced encapsulation efficiency (EE) of these nanocarrier systems are the main constraints that reduce and hinder the antibacterial activity of CEF. Recently, nanogels as a novel class of drug delivery systems have gained substantial attention in biomedicine due to their unique physicochemical properties [9,10,11]. Nanogels are three-dimensional crosslinked networks formed by hydrophilic or amphiphilic polymers through physical or chemical interactions. They are characterized by high LC and EE, excellent biocompatibility, and adjustable drug release profiles [12]. Compared to other nanoscale drug delivery systems such as liposomes, nanogels exhibit distinct advantages: (1) the porous structure enables efficient encapsulation of both hydrophobic and hydrophilic drugs; (2) controlled drug release can be achieved by modulating crosslinking density or environmental responsiveness (e.g., pH, temperature, or enzyme sensitivity); (3) surface functionalization facilitates targeted delivery. For instance, pH-sensitive nanogels maintain structural integrity in the acidic gastric environment (pH 1.5–3.5), shielding drugs from degradation, while undergoing swelling or dissociation in the neutral-to-alkaline intestinal environment (pH 6.5–7.5) to achieve site-specific release [13,14]. These characteristics make nanogels ideal carriers for oral antibiotic delivery systems. The nanogels are critical for constructing orally administered nanogel systems. Carboxymethylcellulose sodium (CMCNa), an anionic polysaccharide derivative, has been extensively utilized as a nanogels matrix due to its high-water solubility, biodegradability, and low toxicity. In gastric acidic conditions (pH < 3), the carboxyl groups (-COO^−^) on CMCNa) chains undergo protonation (-COOH), reducing hydrophilicity and forming a dense hydrogel network that effectively separates encapsulated drugs from gastric acid. CMCNa-based nanocarriers significantly enhance the gastric stability of acid-labile drugs such as insulin or probiotics. Furthermore, the negative surface charge of CMCNa minimizes nonspecific adhesion to intestinal mucosa via electrostatic repulsion, prolonged and extended intestinal retention, and enhanced localized therapeutic effects [15]. On the other side, D-Mannosamine hydrochloride (DMH), a positively charged amino sugar derivative, exhibits targeting specificity attributed to the abundant Type 1 fimbriae on *E. coli* surfaces [16]. These fimbriae express mannose-specific adhesins (FimH) at their tips, which selectively recognize and bind to mannose derivatives. By conjugating DMH onto nanogel surfaces, active targeting of *E. coli* can be achieved through FimH-DMH ligand–receptor interactions [13]. This targeting strategy not only enhances drug enrichment at infection sites but also minimizes collateral damage to commensal microbiota, thereby reducing resistance risks.

**Figure 1:**
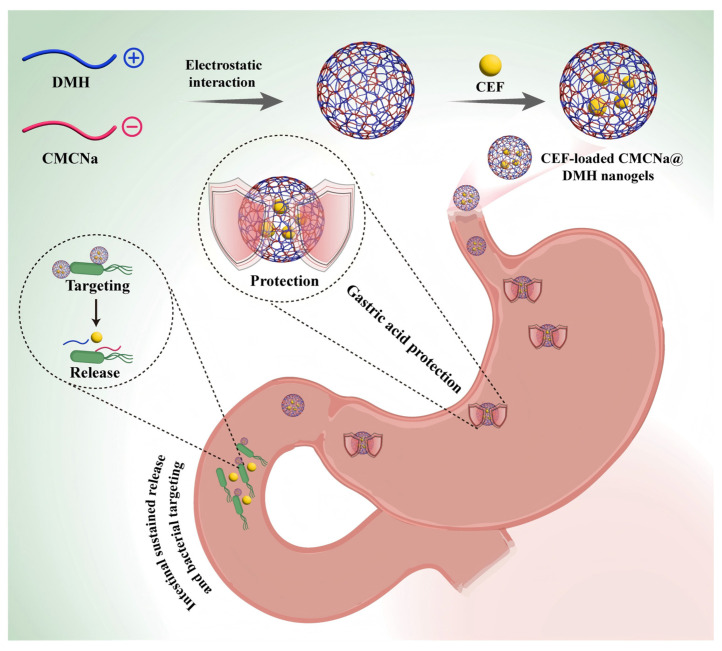
Schematic diagram of a multifunctional nanogel-based delivery system featuring “gastric acid protection, intestinal sustained release, and bacterial targeting”, designed to enhance the antibacterial efficacy of CEF against *E. coli* through sustained release and targeted release.

Given this, a multifunctional nanogels delivery system with “gastric acid protection, intestinal sustained release, and bacterial targeting” was designed, in which the negatively charged CMCNa and positively charged DMH were used as the matrix, and CEF was encapsulated through electrostatic action. The CMCNa component leverages gastrointestinal fluid-responsive behavior to establish dual-phase regulation: a gastric acid-resistant barrier and controlled intestinal release. Simultaneously, DMH mediates active targeting to enhance antibacterial efficacy against *E. coli.* More importantly, the nanogel’s sustained-release properties prolong drug action duration, and reduce dosing frequency (Figure 1). Thus, it is hypothesized that the multifunctional nanogel delivery system with “gastric acid protection, intestinal sustained release, and bacterial targeting” has obvious gastrointestinal fluid-responsiveness and excellent antibacterial activity through sustained and targeted release. The optimal formulation, physicochemical properties, gastrointestinal fluid-responsive performance, stability, and antibacterial activity have been studied systematically. This work aims to improve the antimicrobial activity of CEF against *E. coli* by protecting CEF from gastric acid degradation and achieving a sustained and targeted effect.

## 2. Results and Discussion

### 2.1. Formulation Optimization

LC and EE are often used to screen for the optimal nanoformulation. The larger the LC and EE, the more drugs are encapsulated. The result was entirely the product of random errors, even though it was in the formulation. When LC is at its maximum, the optimal amount and proportion of the nanogels are obtained [17,18]. By determining the relationship between the generated response surfaces and the controllable input parameters, the response surface approach facilitates the investigation and modeling the formulation difficulties and process parameters. It does this by fusing mathematical and statistical methods [19]. To obtain a suitable estimate of the prediction variance across the whole model design space, thirteen experimental runs with three repeated center points were needed. Table 1 displays the trial design and outcomes produced by the Design-Expert tool. Using different concentrations of CMCNa (50, 100, and 200 mg/mL) and DMH (10, 20, and 40 mg/mL) as variables, and LC and EE as evaluation indices, the optimal formula was screened. The concentrations of auxiliary materials (CMCNa and DMH) were too high or too low; the LC and EE of the CEF nanogels will be affected. High concentration of auxiliary materials leads to high weight of the whole CEF nanogels; the calculated LC and EE of CEF nanogels were relatively low. The low concentrations of auxiliary materials make it difficult for CEF to be encapsulated, thus, the LC and EE of CEF nanogels were also relatively low. Therefore, the appropriate concentration of auxiliary materials will get the maximum LC and EE of CEF nanogels. Following the analysis, the quadratic polynomial regression equation linking the LC to three components and the thirteen groups’ findings was shown in Tables S1 and S2 (A: CMCNa; B: DMH):

(1) $$\mathrm{LC}=14.04+1.13\times A+0.4976\times B-0.4714\times \mathrm{AB}-1.64A^{2}-0.8397\times B^{2}$$

The quadratic polynomial regression equation between the EE and three factors was (A: CMCNa; B: DMH):

(2) $$\mathrm{EE}=54.1+1.12\times A+0.6828\times B+0.2951\times \mathrm{AB}-2.9\times A^{2}-2.85\times B^{2}$$

**Figure 2:**
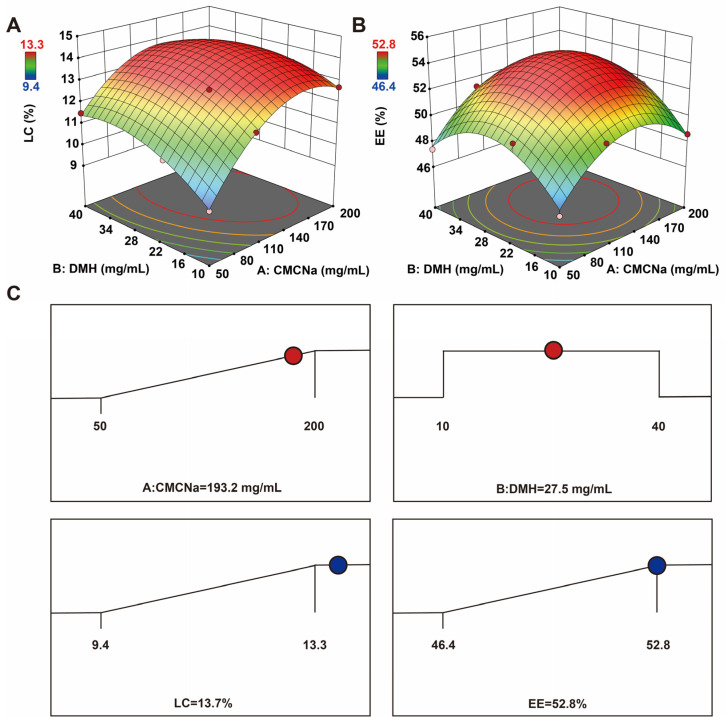
Optimization of CEF nanogels. Three-dimensional arrangement for response surface images of the different concentrations of CMCNa and DMH to LC (**A**) and EE (**B**). (**C**) The optimal formulation predicted by Design-Expert software.

The residual lack of fit was not significant (*p* > 0.05), as evidenced by the highly substantial discrepancies between the different treatments of the LC and EE models (*p* < 0.0001). The regression equation coefficients R^2^, adjusted R^2^, and adjusted R^2^ were 0.9841, 0.9727, and 0.8959 for EE, and 0.9951, 0.9916, and 0.9296 for LC, respectively, reflecting the range of response values. More importantly, |adjusted R^2^ − pre R^2^| < 0.2 (LC and EE), demonstrating that both prediction models were reliable. Three-dimensional response surface images were generated based on the above data (Figure 2A,B). The optimized CEF nanogels, according to Design-Expert software 8.0, were 193.2 mg/mL CMCNa and 27.5 mg/mL DMH. The LC and EE predicted by the software were 13.7% and 52.8%, respectively (Figure 2C). Subsequently, the optimal formulation was verified by producing CEF nanogels with 195 mg/mL CMCNa and 28 mg/mL DMH. The LC and EE of the prepared CEF nanogels were 13.0% ± 1.7% and 52.2% ± 1.0%, respectively. Thus, the optimal preparation method for the CEF nanogels designed by the Box–Behnken response surface technique was accurate and reliable.

### 2.2. Characterization

**Figure 3:**
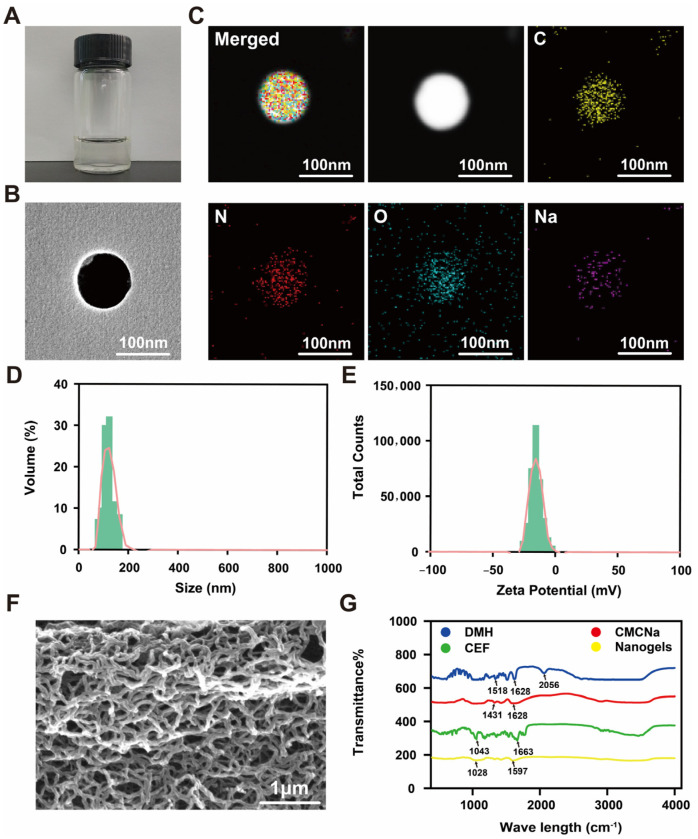
Characterization of CEF nanogels. Appearance (**A**), TEM (**B**); EDS (**C**); size distribution (**D**); Zeta potential (**E**); SEM (**F**); FTIR (**G**).

In recent years, an increasing number of CEF delivery platforms have been used to enhance the antibacterial activity of drugs. For example, to improve the strategy of CEF in the treatment of animal bacterial infection, a novel oily nanosuspension was designed to improve the stability and bioavailability of CEF, reduce muscle irritation, and the cost of production. However, the particle size (≈750 nm) and PDI (≈0.82) of the CEF oily nanosuspension were relatively large, which may reduce the therapeutic efficacy of the CEF. This may be attributed to the preparation method of the CEF oily nanosuspension [5]. The CEF proliposomes prepared by the solid-dispersion method combined with efferent vesicle hydration had an EE of only 63.21% and an LC of only 4.04%. The lower LC and EE greatly reduce the efficacy of the drug and increase the cost of the drug [8]. CEF gelatin microspheres were prepared as a sustained-release formulation using the emulsion chemical cross-linking technique. However, the mean diameter was 8.80 ± 0.78 μm. Large particle size is not conducive to sufficient interaction between the formulation and bacteria, thereby reducing the antibacterial activity of CEF [7]. In this study, a multifunctional nanogels delivery system with “gastric acid protection, intestinal sustained release, and bacterial targeting” was designed by the electrostatic action between CMCNa and DMH. The combination of gastric protection and *E. coli*-targeted delivery using DMH is an innovative strategy that was recently reported in veterinary drug delivery. Thus, the appearance, pH, TEM, mean size, ZP, PDI, SEM, and FTIR spectrum of optimal CEF nanogels were systematically evaluated in this study. The consistent, clear, transparent color of the CEF nanogels in the bottle provided preliminary confirmation of their good hydrogel stability (Figure 3A). The pH of CEF nanogels was 7.0. The CEF nanogels were found to be smooth and spherical by TEM investigation (Figure 3B). Additionally, CEF nanogels’ EDS showed that C, N, O, and Na were evenly distributed throughout the nanogels, which provided ample evidence for the successful construction of nanogels delivery system through the electrostatic action between CMCNa and DMH (Figure 3C). In addition, the mean size, ZP, and PDI of CEF nanogels were 116.9 ± 1.2 nm (Figure 3D), −17.8 ± 0.4 mV (Figure 3E), and 0.14 ± 0.05, respectively, which demonstrated the homogeneous dispersion and nanoscale nature of the CEF nanogels. SEM of freeze-dried CEF nanogels showed a three-dimensional network structure (Figure 3F), and this high specific surface area characteristic may enhance its drug loading ability and drug release performance. The FTIR spectra of CEF, CMCNa, DMH, and CEF nanogels are shown in Figure 3G. Each component’s physicochemical interactions were examined using FTIR spectroscopy. For example, the electrostatic interaction contacts between CMCNa and DMH can result in frequency shifts or absorption peak splitting. The distinctive peaks for DMH at 1518, 1628 and 2056 cm^−1^, CMCNa at 1431 and 1628 cm^−1^, and CEF at 1043 and 1663 cm^−1^ vanished from the spectrum of CEF nanogels, and new characteristic peaks (1028 and 1597 cm^−1^) were visible instead, which may be attributed to the electrostatic action between CMCNa and DMH. Thus, CEF nanogels were successfully prepared by the electrostatic action between CMCNa and DMH.

### 2.3. Stability

**Figure 4:**
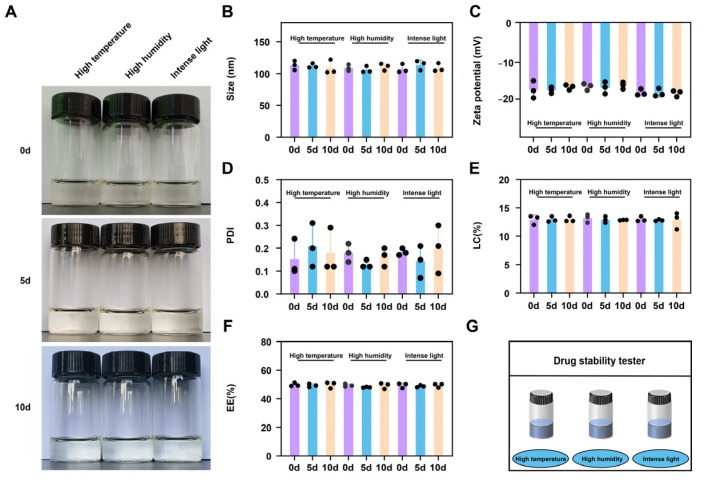
The influence factors test (high temperature, high humidity, and intense light) of CEF-loaded CMCNa@DMH nanogels. Appearance (**A**); size (**B**); ZP (**C**); PDI (**D**); LC (**E**); EE (**F**); schematic diagram of stability of CEF nanogels incubated at 40 °C, 90% ± 5% humidity, and 4500 ± 500 L× for illumination (**G**).

To investigate the stability of CEF nanogels, influencing-factor experiments, which included high temperature (40 °C), high humidity (90% ± 5%), and intense light (4500 ± 500 L×) in the drug stability tester (Figure 4G), were carried out by using their appearance, size, ZP, PDI, EE, and LC as an evaluation indicator. There were no significant differences in appearance (always clear transparent color in the bottle) (Figure 4A), size (about 120 nm) (Figure 4B), ZP (about −18.0 mV) (Figure 4C), PDI (about 0.2) (Figure 4D), EE (about 52%) (Figure 4E), and LC (about 13%) (Figure 4F) on the fifth and tenth days. It is suggested that the prepared CEF nanogels hold satisfactory stability. Thus, the results indicated that nanocarrier systems can effectively improve the stability of drugs.

### 2.4. Gastrointestinal Fluid-Responsive Performances

**Figure 5:**
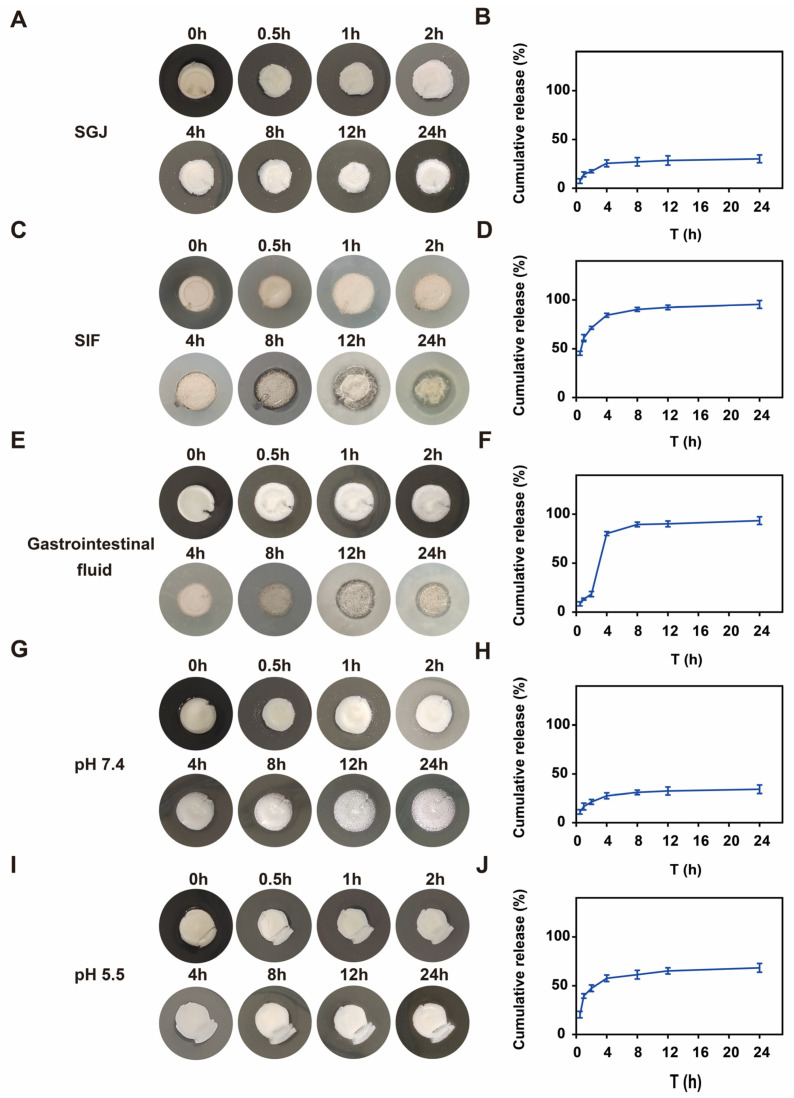
Morphology change and in vitro release of CEF-loaded CMCNa@DMH nanogels in SGJ (**A**,**B**), SIF (**C**,**D**), simulated gastrointestinal fluid (**E**,**F**), pH 7.4 (**G**,**H**), and pH 5.5 (**I**,**J**).

This work comprehensively assessed the CMCNa@DMH nanogels loaded with CEF’s gastrointestinal fluid-responsive performance in different microenvironments (SGJ, SIF, and gastrointestinal fluid; pH 7.4 and pH 5.5). The cefquinome-loaded nanogel exhibited distinct gastrointestinal fluid-responsive drug release profiles, demonstrating differential structural stability and release kinetics under various physiological and pathological environments. The CEF nanogels showed limited drug release in SGF, with only 30.2% ± 4.0% cumulative release over 24 h (Figure 5A,B), whereas, in SIF, a significantly higher release (95.5% ± 4.0%) was observed (Figure 5C,D), indicating inherent enteric solubility. To better mimic gastrointestinal transit, a dynamic release model (2 h in SGF followed by about 20 h in SIF) was employed. During the gastric phase, the CEF nanogels maintained structural integrity with only 18.5% ± 2.7% drug release, consistent with typical gastric emptying time (~2 h). Upon the intestinal phase, rapid structural disintegration occurred, triggering a burst release (93.5% ± 4.0% cumulative release), which confirmed efficient intestinal-targeted delivery (Figure 5E,F). Moreover, the CEF nanogels displayed pH-dependent release behavior in simulated physiological (pH 7.4) and infection microenvironments (pH 5.5). At neutral pH (7.4), 68.3% ± 4.6% of the CEF was released within 24 h (Figure 5G,H), whereas, under weakly acidic conditions (pH 5.5), the release was significantly suppressed (34.3% ± 4.4%) (Figure 5I,J). This pH-responsive characteristic maintains sustained drug release in acidic infection sites, which prolongs local therapeutic effects, and more importantly, accelerates clearance in neutral tissues to minimize systemic toxicity.

### 2.5. In Vitro Antibacterial Activity

**Figure 6:**
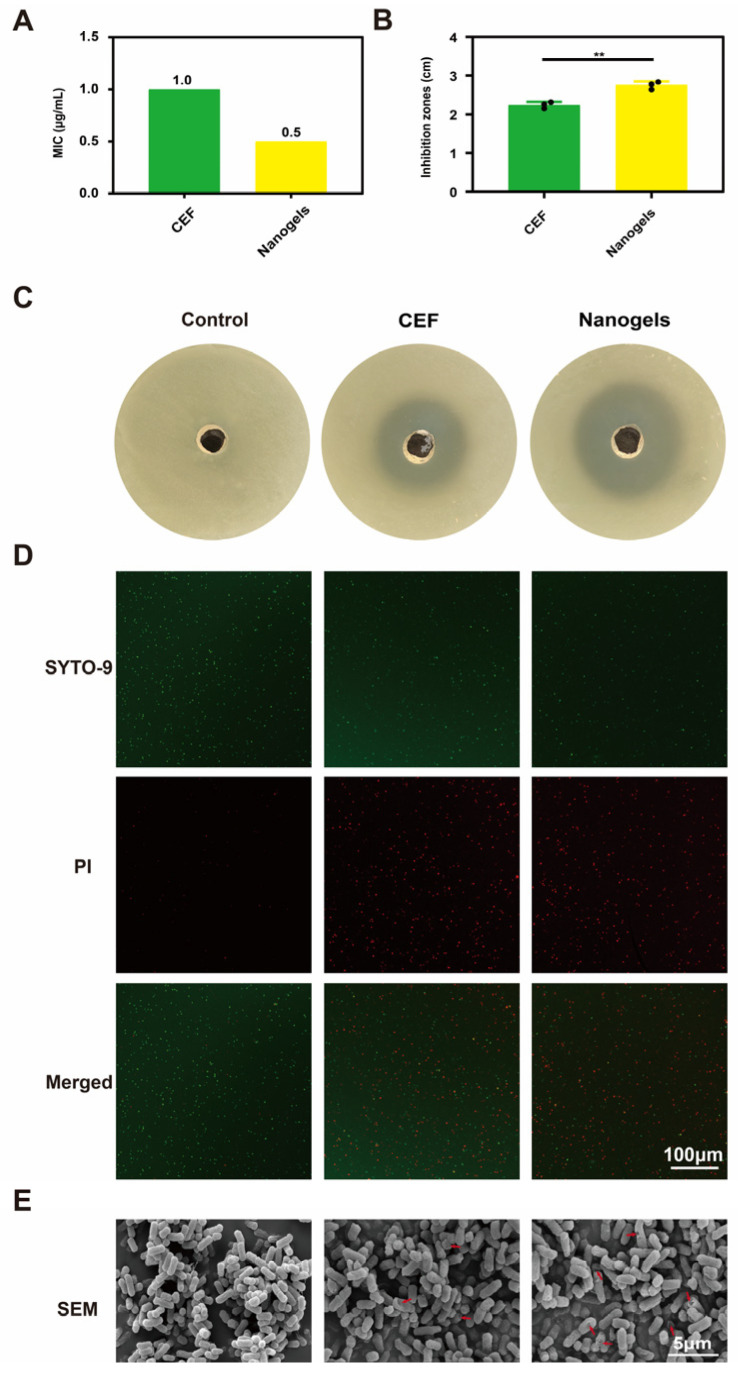
In vitro antibacterial activity of CEF nanogels against *E. coli* isolates. (**A**) MICs; (**B**,**C**) inhibition zones. (**D**) Live/dead bacterial staining images; (**E**) SEM images. (** *p* < 0.01).

The in vitro antibacterial activity of CEF and CEF-loaded CMCNa@DMH nanogels against isolates of *E. coli* was demonstrated in Figure 6. *E. coli* isolates had MICs of 1.0 and 0.5 µg/mL for CEF and CEF nanogels, respectively (Figure 6A). *E. coli* isolates showed inhibitory zones of 2.24 ± 0.07 cm for CEF and 2.75 ± 0.08 cm for CEF nanogels (Figure 6B,C). *E. coli* isolates were thus more susceptible to the antibacterial action of CEF nanogels than CEF. The delivery strategy of nanogels may enhance the antibacterial effectiveness of CEF through targeted and sustained release, according to this dose–response relationship. Additionally, the live/dead bacterial staining kit was used to treat the mixture of *E. coli* isolates with CEF or CEF nanogels. As demonstrated by the increase in dead bacteria (colored red) and decrease in living bacteria (colored green), the results showed that CEF nanogels had more bactericidal efficacy than CEF (Figure 6D). This synergistic effect may originate from the “drug storage effect” of the nanogels delivery system, that is, maintaining the local effective drug concentration through the sustained release characteristics, while the targeting effect of DMH enhances the drug-bacteria interaction. After the *E. coli* isolates were treated with CEF nanogels, SEM showed that the *E. coli* isolates had more holes and lysis. After treatment with CEF nanogels, the cell wall of *E. coli* isolates showed obvious holes and cytoplasm leakage, indicating that nanogels delivery system may synergistically enhance the bactericidal effect of CEF by destroying the integrity of bacterial membrane (Figure 6E). The synergistic mechanism of “structural damage drug penetration” is consistent with previous research reports on enhancing the antibacterial effect of Gram-negative bacteria by nanogels delivery system.

## 3. Conclusions

In this study, a multifunctional nanogel delivery system with “gastric acid protection, intestinal sustained release, and bacterial targeting” was designed for animal-derived diarrhea caused by *E. coli*. Physicochemical characterization proved that the CEF nanogels were successfully prepared and had obvious gastrointestinal fluid-responsiveness and satisfactory stability. Moreover, the prepared CEF nanogels had stronger antibacterial activity against *E. coli* than CEF through sustained and targeted release. Therefore, the multifunctional nanogel delivery system (CEF nanogels) prepared in this study is expected to provide a new nano preparation for animal diarrhea caused by *E. coli*.

## 4. Materials and Methods

### 4.1. Materials

DMH, CEF (content: 97%), simulated gastric juice (SGJ), and simulated intestinal fluid (SIF) were purchased from Macklin (Shanghai, China). CMCNa was obtained from Suzhou Intelligent Manufacturing Research Institute (Suzhou, China). Phosphate-buffered saline (PBS) and tryptone soy broth (TSB) were provided by Dingyuan Biotechnology Co., Ltd. (Alar, China). Shanghai Bestbio Biotechnology Co., Ltd. provided a live/dead illuminated bacterial viability kit (Shanghai, China). The engineering laboratory for the diagnosis and control of animal diseases in Tarim, China, supplied the *E. coli* isolates (The *E. coli* isolates were isolated from piglet manure in a Henan farm; Henan, China).

### 4.2. Formulation of CEF-Loaded CMCNa@DMH Nanogels

The CEF-loaded CMCNa@DMH nanogels, composed of CEF, CMCNa, and DMH, were formulated by electrostatic action. Briefly, CMCNa (50, 100, or 200 mg), DMH (10, 20, or 40 mg), and CEF (1 mg) were added to 1 mL of ultrapure water with stirring to completely dissolve to obtain CMCNa solution, DMH solution, and CEF solution, respectively. Subsequently, the dissolved DMH solution was added dropwise to the CMCNa solution by using magnetic stirring at 800 RPM for one hour. To create CEF-loaded CMCNa@DMH nanogels, the CEF solution was then added dropwise (1 drop/10 s) to the CMCNa/DMH mixed solution while being magnetically stirred for one hour at 800 RPM. The freshly prepared CEF nanogels were kept at room temperature before further usage and tests. Meanwhile, the pH of CEF nanogels was measured. Briefly, 10 mL of ultrapure water was added to 0.1 mL of CEF nanogels, and shook it at 100 rpm for 24 h at room temperature until swelling equilibrium occurs. The pH of the hydrogel was then measured directly by the pH meter (PHG-2081, Shanghai Botuo Instrument Co., Ltd., Shanghai, China).

### 4.3. Box–Behnken Response Surface Analysis

The Design-Expert 8.0 program (State-Ease, Inc., Minneapolis, MN, USA) precisely identified the optimal values of DMH and CMCNa. LC and EE were applied as assessment indices. Table 1 displays factors and values of the Box–Behnken design [19].

### 4.4. Physicochemical Characterization

The surface morphology of CEF-loaded CMCNa@DMH nanogels was characterized using their appearance and TEM. Briefly, the freshly prepared CEF nanogels were observed in a bottle. Additionally, CEF nanogels were diluted 100 times and subjected to an ultrasonic cleaner (BL3-120A, Shanghai Jianglai Biotechnology Co., Ltd., Shanghai, China) for one hour each. Transmission electron microscopy (TEM, JEM-2100Plus, JEOL, Tokyo, Japan) was then used to investigate the CEF nanogels, and energy dispersive spectroscopy (EDS; X-Max N 150; Oxford, UK) was used to determine the elemental analysis. Following the lyophilization of the CEF nanogels using a lyophilizer (FDU-1200, Beijing Songyuan Huaxing Biotechnology Co., Ltd., Beijing, China), scanning electron microscopy (SEM, APREO, Thermo Scientific Inc., Waltham, MA, USA) was used to examine the morphology of the freeze-dried CEF nanogels. Additionally, the mean size, zeta potential (ZP), and polydispersity index (PDI) of CEF-loaded CMCNa@DMH nanogels were measured using the Zetasizer ZX3600 (Malvern Instruments, Malvern, UK). Finally, Fourier transform infrared (FTIR) spectra of CEF, CMCNa, DMH, and CEF-loaded CMCNa@DMH nanogels were analyzed using an FTIR spectrophotometer (Nicolet iS50, Thermo Scientific Inc., Waltham, MA, USA), respectively.

### 4.5. Gastrointestinal Fluid-Responsive Release

In this study, the gastrointestinal fluid-responsiveness of CEF-loaded CMCNa@DMH nanogels in different microenvironments (SGJ, SIF, and gastrointestinal fluid; pH 7.4 and pH 5.5) was evaluated. Briefly, the lyophilized CEF-loaded CMCNa@DMH nanogels were initially placed in SGJ (2 h) and subsequently in SIF (8 h) to assess the gastrointestinal fluid-responsive performance. Simultaneously, the lyophilized CEF-loaded CMCNa@DMH nanogels were initially placed in pH 7.4 and pH 5.5. At various time points, the morphological variations were observed in different microenvironments. Concurrently, the CEF-loaded CMCNa@DMH nanogels’ cumulative curves in various microenvironments were shown [20].

### 4.6. Stability Evaluation

The stability of CEF-loaded CMCNa@DMH nanogels was evaluated using influence-factor studies, which comprised high temperature (40 °C), high humidity (90% ± 5%), and bright light (4500 ± 500 L×) at the Drug stability tester (WD-2A, Guangzhou Huruiming Instrument Co., Ltd., Guangzhou, China). The CEF nanogels were put in a container and incubated for 10 days at 40 °C, 90% ± 5% humidity, and 4500 ± 500 L× for illumination to perform the high temperature test. In order to evaluate the differences in their appearance, mean size, ZP, PDI, LC, and EE, samples were taken on the fifth and tenth days [14].

### 4.7. In Vitro Antibacterial Activity Studies

#### 4.7.1. Broth Macrodilution Method

The minimum inhibitory concentrations (MICs) of CEF and CEF-loaded CMCNa@DMH nanogels against *E. coli* isolates were studied using the broth microdilution method [14]. Briefly, serial dilutions of CEF or CEF nanogels in TSB broth were prepared. A final bacterial concentration of 1 × 10^6^ CFU/mL was then attained for each tube using *E. coli* isolates. As a negative control, PBS was employed. The lowest quantity that, after 24 h of incubation at 37 °C, inhibited the visible growth of bacteria was determined to be the MICs of CEF or CEF nanogels against *E. coli* isolates.

#### 4.7.2. Bacterial Inhibition Zones

The inhibition zones of CEF and CEF nanogels against *E. coli* isolates were estimated [14]. Briefly, an aseptic plate was filled with 15 milliliters of agar medium. Following the solidification of the agar, 0.1 mL of bacterial fluid containing 1 × 10^6^ CFU/mL of *E. coli* isolates was added, along with an additional 5 mL of agar medium. Once set, the appropriate straw was used to create holes in the solid agar. Then, either 50 µL of CEF or CEF nanogels were introduced. As a negative control, PBS was employed. The size of the inhibitory zones was measured and recorded after the *E. coli* isolates were cultured for 24 h at 37 °C in an incubator with 5% CO_2_.

#### 4.7.3. Analysis of Live/Dead Bacterial Staining

Furthermore, the live/dead bacterial staining method was used to systematically assess the antibacterial properties of CEF and CEF nanogels [15]. In short, *E. coli* isolates were mixed with CEF or CEF nanogels. After a two-hour incubation period, all samples were processed using the live/dead fluorescent bacterial viability kit. Eventually, 5 μL of the bacterial suspension that was placed onto the slide was examined using a laser confocal microscope (A1RHD25&-SIM, Nikon, Tokyo, Japan). In this investigation, physiological saline served as a negative control.

#### 4.7.4. Morphological Analysis

SEM was used to observe the morphological changes in *E. coli* isolates treated with CEF and CEF nanogels [15]. In conclusion, *E. coli* isolates (1 × 10^6^ CFU/mL) were cultivated in triplicate on a cover glass for 24 h at 37 °C using CEF or CEF nanogels (all 1 × MIC). Subsequently, 2.5% glutaraldehyde was used to fix the samples for two hours at 4 °C. The sample was dried using ethanol concentrations of 30%, 50%, 70%, 90%, 95%, and 100% for a total of 20 min after the surfaces had been cleaned twice for 15 min each. Subsequently, each sample was gold sputtered and coated by a fully automatic ion sputtering instrument (Cressington 108Auto, Beijing Zhongxing Bairui Technology Co., Ltd., Beijing, China) for 120 s with a working current of 25 mA. The final gold spray thickness of each sample was about 10 nm, then analysed using SEM. PBS was employed as a negative control.

### 4.8. Statistical Analysis

The experimental data are expressed as Mean ± S.D. and analyzed by one-way ANOVA using the SPSS 19.0 software, followed by Tukey’s post-hoc test for multiple comparisons. The sample size (n = 3/group) was determined based on a power analysis (α = 0.05, power = 0.80), ensuring adequate sensitivity to detect group differences. Statistical significance was set at * *p* < 0.05.

## Figures and Tables

**Table 1:** Single-factor experimental design and responses for CEF nanogels.

Run	CMCNa (mg/mL)	DMH (mg/mL)	LC (%)	EE (%)
1	50	10	9.4	46.4
2	200	10	12.7	48.6
3	50	40	11.5	47.4
4	200	40	12.8	50.5
5	50	20	10.8	50.3
6	200	20	13.3	51.5
7	100	10	12.0	50.3
8	100	40	13.1	51.3
9	100	20	13.2	52.8
10	100	20	13.2	52.8
11	100	20	13.2	52.8
12	100	20	13.2	52.8
13	100	20	13.2	52.8

## Data Availability

The data used to support the findings of this study are available from the corresponding author upon request.

## Supplementary Materials

The following supporting information can be downloaded at: https://www.mdpi.com/article/10.3390/gels11070503/s1, Table S1. ANOVA of the LC model. Table S2. ANOVA of the EE model.
