# Supplementary material for: Gastric Acid-Protective and Intestinal Targeted Nanogels Enable Anti-Bacterial Activity of Cefquinome
Source: Gels. 2025 Jun 27;11(7):503. doi: 10.3390/gels11070503 (PMC12294563; doi:10.3390/gels11070503)
Supplement: Supplementary file 1 [file gels-11-00503-s001.zip › gels-3690021-supplementary.pdf]

Supplementary Materials

Gastric Acid-Protective and Intestinal Targeted Nanogels Enable Anti-Bacterial Activity of Cefquinome

Xianqiang Li [1,†], Tianhui Wang [2,†], Shuo Han [2,†], Jinhuan Liu [2,3], Xiuping Zhang [2], Zhiqiang Zhou [4],
Ali Sobhy Dawood [5] and Wanhe Luo [2,*]

1 College of Biomedicine and Health, Anhui Science and Technology University, Chuzhou 233100, China; lixianqiang89@sina.com
2 Engineering Laboratory for Tarim Animal Diseases Diagnosis and Control, College of Animal Science and Technology, Tarim University, Alar 843300, China; w3505506223@163.com (T.W.); 15645977635@163.com (S.H.); liujinhuan0830@163.com (J.L.); zxpkdy@126.com (X.Z.)
3 College of Veterinary Medicine, Sichuan Agricultural University, Chengdu 611130, China
4 Instrumental Analysis Center, Tarim University, Alar 843300, China; zhou2011519@sina.com
5 Infectious Diseases, Faculty of Veterinary Medicine, University of Sadat City, Sadat City 32897, Egypt; ali.dawood@vet.usc.edu.eg
* Correspondence: luowanhe0728@163.com
† These authors contributed equally to this work.

Table S1. ANOVA of the LC model.

Source	Sum of Squares	df	Mean Square	F-value	P-value	
Model	17.18	5	3.44	284.78	<0.0001	significant
A-CMCNa	7.54	1	7.54	625.20	<0.0001	
B-DMH	1.46	1	1.46	121.04	<0.0001	
AB	0.9564	1	0.9564	79.26	<0.0001	
A^2	5.58	1	5.58	462.43	<0.0001	
B^2	1.46	1	1.46	121.28	<0.0001	
Residual	0.0845	7	0.0121			
Lack of Fit	0.0845	3	0.0282			
Pure Error	0.0000	4	0.0000			
Cor Total	17.27	12				
Std.Dev	0.1099					

Mean	12.43
CV%	0.8837
R^2	0.9951
Adjiusted R^2	0.9916
Predicted R^2	0.9296
Adeq Precision	53.1766

Table S2. ANOVA of EE model.

Source	Sum of Squares	df	Mean Square	F-value	P-value	
Model	56.18	5	11.24	86.42	<0.0001	significant
A-CMCNa	7.35	1	7.35	56.52	0.0001	
B-DMH	2.75	1	2.75	21.15	0.0025	
AB	0.3750	1	0.3750	2.88	0.1333	
A^2	17.50	1	17.50	134.59	<0.0001	
B^2	16.90	1	16.90	129.99	<0.0001	
Residual	0.9101	7	0.1300			
Lack of Fit	0.9101	3	0.3034			
Pure Error	0.0000	4	0.0000			
Cor Total	57.09	12				
Std.Dev	0.3606					
Mean	50.79					
CV%	0.7099					
R^2	0.9841					
Adjusted R^2	0.9727					
Predicted R^2	0.8959					
Adeq Precision	25.7157					
